# The Occurrence and Diversity of Waterborne Fungi in African Aquatic Systems: Their Impact on Water Quality and Human Health

**DOI:** 10.3390/ijerph14050546

**Published:** 2017-05-20

**Authors:** Nontokozo M. Magwaza, Edward N. Nxumalo, Bhekie B. Mamba, Titus A. M. Msagati

**Affiliations:** Nanotechnology and Water Sustainability Research Unit, College of Science Engineering and Technology, University of South Africa, Roodepoort, 1710 Johannesburg, South Africa; nomagwazanontokozo@gmail.com (N.M.M.); nxumaen@unisa.ac.za (E.N.N.); mambabb@unisa.ac.za (B.B.M.)

**Keywords:** aquatic fungi, secondary metabolites, human health

## Abstract

Currently, there is a worldwide growing interest in the occurrence and diversity of fungi and their secondary metabolites in aquatic systems, especially concerning their role in water quality and human health. However, this concern is hampered by the scant information that is available in the literature about aquatic fungi and how they affect water quality. There are only few published reports that link certain species of aquatic fungi to human health. The common aquatic fungal species that have been reported so far in African aquatic systems belong to the hyphomycetes kingdom. This paper thus aims to survey the information about the occurrence and factors that control the distribution of different species of fungi in African aquatic systems, as well as their effect on water quality and the possible metabolic pathways that lead to the formation of toxic secondary metabolites that are responsible for the deterioration of water quality. This review will also investigate the analytical and bioanalytical procedures that have been reported for the identification of different species of waterborne fungi and their secondary metabolites.

## 1. Introduction

According to the World Health Organization (WHO), water that can be regarded as safe and fit for human consumption is that which cannot cause any significant health hazard when consumed and that which has microbial, chemical and physical characteristics that meet WHO guidelines [1].

Among the most serious concerns with regard to water quality is the presence of and contamination due to microbial pathogens in water. This has prompted water utilities, stakeholders in water issues and all other sectors that are responsible for the supply and distribution of drinking water to come up with strategies to protect consumers from waterborne diseases, as well as to ensure that the water that reaches the public does not harbor any pathogenic microorganisms. According to the few available reports, the monitoring of microbial pathogens in most drinking water systems is dependent on the monitoring of indicator microorganisms (bacteria), especially measures of the total coliforms and *Escherichia coli* [2,3,4,5]. However, in reality, coliforms by themselves may not necessarily be a health hazard, but act as an indicator for the presence of pollution in water systems.

It is well understood that there are many microbes that are present in aquatic systems that pose a real health threat to consumers and which may not be indicated by the presence of coliforms. For example, the presence of harmful algal blooms, water viruses, protozoans and fungi may not be indicated by coliform. For a thorough water monitoring scheme, it may be desired that the procedures for water monitoring for the presence of harmful microorganisms go beyond the monitoring of coliforms. This issue carries even more weight at the present time, as there is worldwide concern for the dwindling of water resources, which is further stressed by high pollution loads as well as microbial contamination.

## 2. Aquatic Fungi in African Waters

Only scant information is available on the growing and spreading of aquatic fungal species in some parts of the world, but even fewer studies have been carried out to date on African aquatic systems. In African waters, the common aquatic fungi that have been reported are those that fall under the kingdom Hyphomycetes, which have been found attached to submerged plants, and also some conidia attached to conidiophores which have been isolated from submerged leaves. The link between their occurrence in both water and aquatic plants is based on the fact that fungal species that grow in streams tend to decay in plant leaves and are then transported from water streams to plants [6].

Previous studies on fungi in South African waters have revealed the presence of mainly aquatic hyphomycetes [7,8]. In Nigeria, the presence of dominant aquatic hyphomycetes was also reported as *Flagellospora curvula* [9]; however, these fungal species reported in Nigeria appeared to be different from the ones reported in South African aquatic systems [6,7,9,10]. Aquatic spores isolated from scums in Nigeria showed the presence of aquatic hyphomycetes and aquatic basidiomycetes [11]. The morphology of the spores reported by Ingold [11] in Nigeria corresponded to the species of *Tetrachaetum* and *Alatospora.* The species of aquatic basidiomycetes are normally produced asexually, parallel to the species of aquatic hyphomycetes [9].

Fungal diversity has also been recorded from brackish and saline water in Egypt. This is the most frequently studied African country; aquatic fungi from species of basidiomycetes, anamorphic fungi and ascomycetes have been reported in various Egyptian lakes. Generally, more reports about aquatic fungi in Egyptian aquatic systems have centred on the diversity of species isolated from brackish lakes, while a few have reported on saline lakes. The diversity trend has been observed to decrease in Egyptian saline water systems; a number of factors contributed to this trend, with the increase of salinity being the major factor [12]. The samples collected from various water sources in Egypt have shown that the most abundant aquatic fungal genera are *Saprolegnia* and *Pythium*, and they are represented by various species [13].

Water systems are known to be contaminated by both terrestrial and zoosporic fungi, and some terrestrial fungal species are translocated from the soil into water and end up growing in the water just like other aquatic fungal species [14]. The movement of some terrestrial fungi into fresh water systems is through animals, plants and soil [15]. The other dominant genus is *Allomyces*, while the abundant species of zoosporic fungi in surface waters such as dams, ponds, rivers and lakes in Egypt are *Ditcyuchus sterile* and *Aphanomyces laevis* [16].

Only a few species have been found to colonize water hyacinth plant leaves such as Eichhornia. Three aquatic hyphomycetes, namely *Alatospora acuminate*, *Triscelophorus monosporus*, and *Tetracladium marchalianum* from the class Eichhornia, have been reported to survive in both summer and winter conditions. Some aquatic hyphomycetes are believed to produce inhibitor compounds which prevent fungi from colonizing plants [17]. Other aquatic fungi which are mainly endophytic are known to produce alkaloids as protective compounds, and some of these endophytic fungi are derived from the genus *Claviceps* [18].

The fungal species that are isolated from plants tend to produce secondary metabolites which are then released in the wood and are responsible for the breakdown of plant tissue. Both primary and secondary metabolites have been reported to break down polymers including carbohydrates as a source of nutrients for these fungal species. The aquatic fungal species that have been reported as being capable of degrading lignin and decaying wood are basidiomycetes. Furthermore, mitosporic fungi and some ascomycetes have aquatic properties, which have also been reported to be responsible for plant decay by lowering the concentration of the total amount of phosphorus, nitrogen and chemical oxygen [19].

## 3. The Effect of Aquatic Fungi in Seafood Consumed by Humans

Aquatic fungi and water molds from freshwater sources have been reported to be responsible for the infection of aquatic species [20,21,22,23,24,25]. The presence of toxic aquatic fungi in water tends to infect aquatic animals, including those that are consumed by humans. These fungal species not only affect fish but also fish eggs [7,26,27,28]. The infected fish can be seen by observing the change in their physical appearance and inability to produce a protective layer due to the damage caused by the toxic substances secreted by fungi [29,30]. Various fish species have been reported to have the disease called epizootic ulcerative syndrome (EUS), which is one of the most dangerous diseases in aquatic ecosystems on the African continent. This disease is caused by the water mold *Aphanomyces invadans* [21]. Among the highest number of infected fish are found in African aquatic systems, and some of the fish species are of economic importance [31]. There are many harmful water molds associated with the infection of fish and other aquatic animals. *Saprolegnia* is one of these molds, which is responsible for the infection of fish skin and leaves minimal chances for recovery [30,32,33,34,35]. A study conducted by Mastan found two genera of water molds which infect aquatic animals, namely *Saprolegnia* and *Achlya*; the most affected aquatic animals were mainly fish species of *Channa*, *Heterioprestis*, *Mystus* and *Labeo* [24]. Eli [36] reviewed various fungal infections on African fish which include Saprolegniasis, Dermal Mycoses; Branchiomyces infections, Systemic Mycoses and Dermocystidium. These fungal infections in fish have not been well studied in Africa, and need more attention in order to protect humans from consuming infected seafood.

The investigation of fungal infections reported in previous studies indicated a high rate of fish mortality [21,23,26,32,36,37]. Through the comparison of infected fish species, investigations have been conducted worldwide (mostly in India), but few studies have been conducted in African countries. Aquatic fungi also causes shrimp diseases, including those that cause changes in skin colour and infections of organs [38]; this was verified by the observations of their secondary metabolites in the infected areas.

Recently, Kumari [39] conducted an investigation on fungal infections in various fish species which included *Gibelion catla*, *Channa striatus*, *Chitala chitala*, *Labeo rohita*, *Channa marulius* and *Carassius auratus.* The results and observations from the abovementioned study suggested that October was the month in which lot of fish were found to be infected with about 25 fungal species. A similar study was previously conducted by Siddique [40] in Bangladesh, where researchers found a diversity of fungi in various types of fish. The similarity between these studies is the month that shows more infections in fish, which in both studies was found to be between October, November and December. Most of the fungal species were found to infect both the interior and exterior parts of aquatic animals such as the head, eyes, gills, fins and abnormal parts. The reported fungal species which were responsible for infecting organs included *Aspergillus* spp., *Penicillium* spp., *Rhizopus* spp., *Blastomyces* spp., and *Alternaria* [41]. In some of the fish species, their tissues were found to be infected with *Aspergillus* species, while *Mucor* spp. were isolated from the gills, abnormal body parts and fins [42].

Nigeria has been reported to have many cases of infected fish due to the presence of fish rearing and fish farming [20]. Fish in Gauteng Province, South Africa, have also been found to suffer from the infection called branchiomycosis caused by two fungal species; *Branchiomyces sanguinis* and *Branchiomyces demigrams*, which grow at temperatures between 25 and 32 °C [36]. These fungal species cause fish infections such as gill rot, which causes gill tissues to become unable to uptake oxygen [43]. The infected areas become necrotic and brownish-grey. Mycotic infections in ponds can lead to epidemics in other fish ponds and this may have very negative implications for fish consumers.

## 4. Human Exposure to Toxic Aquatic Fungi

### 4.1. Direct Toxicity to Humans

Some water sources are contaminated with pathogenic aquatic fungal species. Humans may be directly exposed to fungal toxins either through ingestion of contaminated water and seafood (fish, prawns, *Spirulina*, etc.), or by ingestion of plant products (vegetables and fruit) irrigated using contaminated water. Some of the symptoms of infections due to aquatic fungal species include skin irritation and immunosuppression infections [20,29,33,40]. An investigation conducted in hospitals for patients who might have been affected by contaminated water showed that water in hospitals is contaminated with fungi which contribute to various diseases in humans [44]. In the study by Göttlich et al. [44], the harmful aquatic fungal species *Aspergillus fumigatus* was isolated from patients who were suffering from immunosuppression infections and organ failure. The confirmation of the pathogenic fungi in patients was conducted by comparing the genotypes of the species and the genotype of a microorganism isolated from the patient [4,45].

### 4.2. Indirect Toxicity to Humans

**Figure 1:**
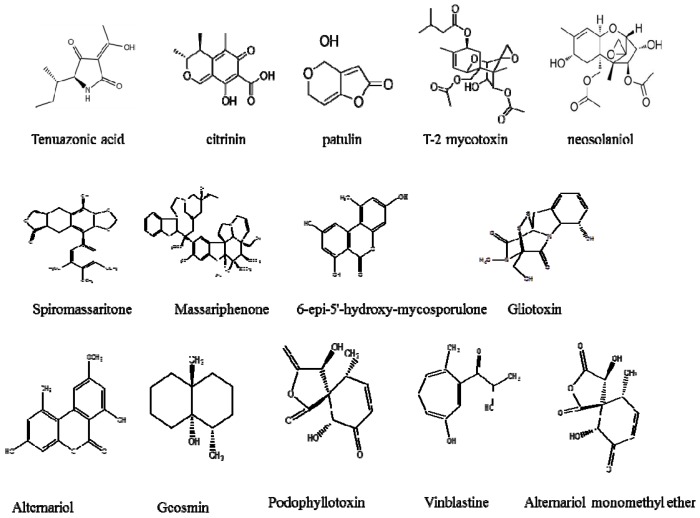
The toxic metabolites produced by aquatic fungal species.

**Figure 2:**
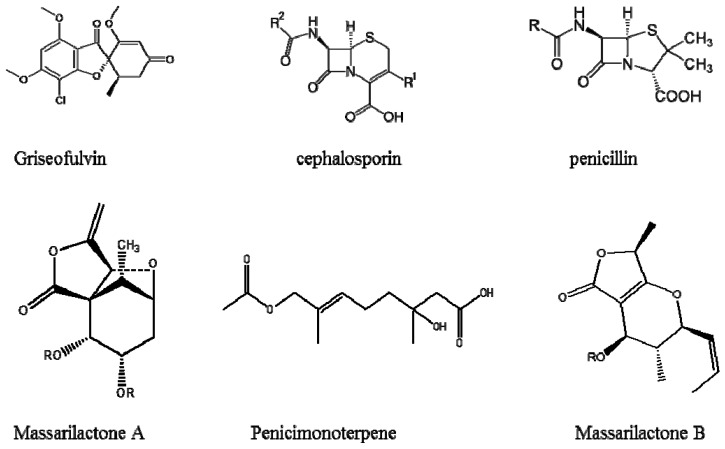
Beneficial metabolites from aquatic fungal species.

**Figure 3:**
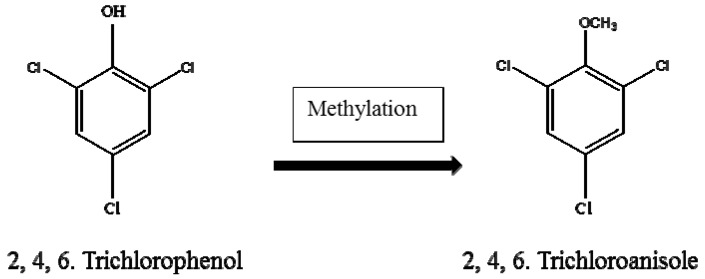
Schematic presentation of the methylation of **2**, **4**, **6** trichlorophenol to **2**, **4**, **6** trichloroanisole.

Humans are easily exposed to fungal contaminations through ingestion of adulterated food, which may result in various diseases and infections. Farmers normally use surface water for watering crops such as vegetables and fruits. Tomatoes have been reported to be very easily infected with fungi. *Alternaria alternate* is the most frequently detected fungus in tomatoes, which causes black molds during growth and even post-harvest [46,47]. Decay of tomatoes is normally caused by various dangerous secondary metabolites that are generated by fungi during plant growth. Harwig et al. [48] isolated various fungal species including *Alternaria alternate*, *Penicillium expansum* and *Fusarium sulphureum*, which produce mycotoxins such as tenuazonic acid, citrinin, patulin, T-2 toxins (trichothecene mycotoxins) and neosolaniol (Figure 1, Figure 2 and Figure 3). Humans are not only exposed to toxic fungal species in agricultural commodities but also those found in seafood, for example fish and prawns. Aquatic fungi in surface water infect aquatic animals, causing infections such as lobomycosis, acute ulceration (ulcerative dermal necrosis) and gill rot in fish [29,33,34,35,49,50].

### 4.3. Aquatic Fungal Secondary Metabolites

Freshwater fungi produce a variety array of antimicrobial metabolites, which help them to compete against other microorganisms [51,52]. However, some of them produce compounds which are responsible for inflammation and chronic diseases [53]. Numerous groups of secondary metabolites and bioactive compounds have been isolated from fungal strains collected from food and water [53,54,55,56]. Some of these compounds are deleterious, while others are beneficial to humankind (Figure 1 and Figure 2). Common fungal species such as *Penicillium* spp. have been found in various water sources [4,44,45]. These species are known to produce harmful metabolites in food which cause negative health effects in humans, though they also produce some useful metabolites, for example cephalosporin, griseofulvin and penicillin which are used as antibiotics. *Tolypocladium* species produce useful metabolites for organ transplants [57]. Numerous studies have been conducted to investigate the secondary fungal metabolites in drinking water. *Penicillium* spp. together with *Aspergillus* spp. produce toxic organic compounds in seafood which cause allergies, asthma and various other infections [4].

Fungal metabolites in drinking water can cause a change in the taste and odour of water [4,45,58]. The change in taste and off-flavour occurs through the methylation process of organic compounds. The microbial methylation process involves the conversion of **2**, **4**, **6**, trichlorophenol to **2**, **4**, **6** trichloroanisole (Figure 3). These compounds (**2**, **4**, **6**, trichlorophenol and **2**, **4**, **6** trichloroanisole) can cause various infections in humans, and are well known to cause off-flavour in foods and drinks [59]. The microbial methylation process occurs during the treatment and distribution of drinking water. Microbial methylation of **2**, **4**, **6**, trichlorophenol to the off-flavour compound **2**, **4**, **6** trichloroanisole causes strong odours in drinking water. The concentration of **2**, **4**, **6**, trichlorophenol in unchlorinated water is lower, while high amounts are found in chlorinated water, and is thus mostly detected in water distribution systems. Humans become exposed to these compounds through inhalation of contaminated air and consumption of contaminated water and food [60].

## 5. Methods for the Isolation of Aquatic Fungi from African Water Systems

The method that is generally applied to isolate and identify fungal species in African water systems depends mainly on the hyphae structure and colour, and involves the use of malt extract agar and distilled water in Petri dishes [6,9,17,61,62]. The mycelium formed in the plate consists of branched hyphae; the branching pattern is then used to identify the species of fungi using a microscope [7]. The diverse colonies in the agar plate represent the fungal species present in the sample. The colour of the pure colony has been observed to change the colour of the entire medium and, after the colony had been kept for 15 days at 30 °C in a Petri dish filled with distilled water, conidia were produced rapidly and abundantly [6].

Ten aquatic hyphomycetes have been isolated from South African water systems through filtering using a 8-μm pore size. These aquatic hyphomycetes were observed in lactophenol cotton blue [62] and identified according to the morphology of the spores [7].

For the recovery of aquatic phycomycetes from water samples collected in sampling bottles containing sesame seeds, samples together with the sesame seeds are normally poured in Petri dishes and left overnight at room temperature around 20 ± 2 °C. After 24 h, the colonized flowering plant seeds are moved to other Petri dishes which contain either sterilised filtered lake water or distilled water and crystalline penicillin for the prevention of bacterial contamination. The Petri dishes are usually incubated for 3 to 5 weeks, and the resulting aquatic phycomycetes are purified on glucose peptone agar medium [13]. All of the previous studies report the same protocol for the isolation of fungi in African water systems. The culture plate and incubation temperature depend on the particular species of fungi. *Aspergillus fumigatus* can be accurately isolated from the culture plate incubated at 30 °C [45].

The culture plate used for each source of water is not the same. A variety of fungal species has been observed in yeast malt extract-glucose agar (YMGA) medium for the detection of fungi in surface water. However, the water-matrix composition was found to differ in spring water and groundwater, although they both showed the diversity of fungi in dichloran rose bengal chlortetracycline (DRBC) medium [4]. Nowadays, there are many culture media designed for fungal growth. For the isolation and enumeration of fungi, the most commonly used culture media are DRBC, dichloran 18% glycerol agar (DG-18), potato dextrose agar (PDA), Sabouraud Dextrose Agar (SDA) and malt extract agar (MEA).

## 6. Methods for the Analysis of Fungal Metabolites

Both classical and modern techniques have been used for the identification of fungal groups/classes/species. Single colonies are isolated from the culture plate for mycological analysis, where they first undergo a staining process with Gram stain or other staining chemicals for better observation of the fungal morphology [22]. The advantages of using classical methods are that the methods are easy to use, they have an acceptable level of accuracy and sensitivity, and they are affordable. However, these methods do not provide additional information about the size and chemical properties of the fungal species, therefore the information on the magnification and resolution of the light microscope is of limited value. Many modern methods are being used for the identification of fungal metabolites; these methods provide detailed chromatograms or spectra for inorganic and organic chemical compounds. These are used for the complete analysis of the analyte of interest, and are highly reliable and faster than conventional identification methods. Satisfactory results are only obtained from the samples that are extracted with suitable extraction methods. There are different extraction methods for metabolite compounds such as liquid-liquid extraction, solid phase extraction, and superficial fluid extraction, although each extraction method is used for a specific set of samples.

A large number of instruments are used as analytical tools for metabolomics studies, such as gas chromatography mass spectrometry (GC-MS), liquid chromatography mass spectrometry (LC-MS), nuclear magnetic resonance (NMR), matrix assisted laser desorption/ionization mass spectrometry (MALDI-MS), and capillary electrophoresis mass spectrometry (CE-MS). However, not all the analytical techniques produce reliable results. These techniques are different in their properties and technological level. Some of them provide the reproducible molecular fragments that are an integral tool for metabolite identification.

## 7. Conclusions

The samples of water and plants that have been investigated in African water systems have shown an abundance of aquatic hyphomycetes species that were identified under the microscope by their morphological characteristics. The use of only one method for the screening of aquatic fungi species in water does not provide enough information about the metabolites or any toxic compounds produced by fungi. Some authors have drawn attention to the presence of aquatic fungi in Africa, but few researchers have actually taken the investigation forward. In African countries, no effort is being made to study the effect of aquatic fungi associated with plants and seafood. However, fungal infections (*Mycosis*, *Saprolegnia*) of the fish in commercial fish farms can have negative economic impacts and thus the industry will not attract investors. Future work on aquatic hyphomycetes must be done to investigate the presence of any toxic and useful aquatic fungal metabolites. Recently, there have been a number of published papers from other countries using various methods and techniques for the analysis of aquatic fungal species in water systems, plants and aquatic animals, involving the use of traditional and molecular methods in order to provide results that confirm the presence of fungi in water. The improvement of water quality may protect aquatic animals from microbial infection, which will then reduce the exposure of humans to contaminated food.
